# The relative expression of miR-31, miR-29, miR-126, and miR-17 and their mRNA targets in the serum of COVID-19 patients with different grades during hospitalization

**DOI:** 10.1186/s40001-021-00544-4

**Published:** 2021-07-13

**Authors:** Reza Keikha, Seyed Mohammad Hashemi-Shahri, Ali Jebali

**Affiliations:** 1 Infectious Diseases and Tropical Medicine Research Center, Resistant Tuberculosis Institute, Zahedan University of Medical Sciences, Zahedan, Iran; 2 Department of Pathology, Faculty of Medicine, Zahedan University of Medical Sciences, Zahedan, Iran; 3 Department of Medical Nanotechnology, Faculty of Advanced Sciences and Technology, Tehran Medical Science, Islamic Azad University, Tehran, Iran

**Keywords:** miRNAs, mRNA, COVID-19, Hospitalization

## Abstract

**Background:**

The aim of this study was to evaluate the expression of four up/down-regulated inflammatory miRNAs and their mRNA targets in the serum samples of COVID-19 patients with different grades. Also, we investigated the relative expression of these miRNAs and mRNAs during hospitalization.

**Methods:**

In this cross-sectional study, 5 mL of blood sample were taken from COVID-19 patients with different grades and during hospitalization from several health centers of Yazd, Tehran, and Zahedan province of Iran from December 20, 2020 to March 2, 2021. The relative expression of miRNAs and mRNAs was evaluated by q-PCR.

**Results:**

We found that the relative expression of hsa-miR-31-3p, hsa-miR-29a-3p, and hsa-miR-126-3p was significantly decreased and the relative expression of their mRNA targets (*ZMYM5*, *COL5A3*, and *CAMSAP1*) was significantly increased with the increase of disease grade. Conversely, the relative expression of hsa-miR-17-3p was significantly increased and its mRNA target (*DICER1*) was significantly decreased with the increase of disease grade. This pattern was exactly seen during hospitalization of COVID-19 patients who did not respond to treatment. In COVID-19 patients who responded to treatment, the expression of selected miRNAs and their mRNA targets returned to the normal level. A negative significant correlation was seen between (1) the expression of hsa-miR-31-3p and *ZMYM5*, (2) hsa-miR-29a-3p and *COL5A3*, (3) hsa-miR-126-3p and *CAMSAP1*, and (4) hsa-miR-17-3p and *DICER1* in COVID-19 patients with any grade (*P*  <  0.05) and during hospitalization.

**Conclusions:**

In this study, we gained a more accurate understanding of the expression of up/down-regulated inflammatory miRNAs in the blood of COVID-19 patients. The obtained data may help us in the diagnosis and prognosis of COVID-19.

*Trial registration*: The ethics committee of Zahedan University of Medical Sciences, Zahedan, Iran. (Ethical Code: IR.ZAUMS.REC.1399.316) was registered for this project.

## Introduction

Coronavirus 2019 (COVID-19) is a worldwide pandemic disease with near 2% mortality [1]. The disease is caused by severe acute respiratory syndrome coronavirus 2 (SARS-CoV-2) and mainly affects the lungs and causes acute respiratory distress syndrome (ARDS) [2]. In COVID-19, there are significant changes in the function and morphology of lung endothelial cells, disruption of intercellular connections, and loss of the basal membrane [3]. From a molecular point of view, the S protein of SARS-CoV-2 binds to the angiotensin-converting enzyme 2 (ACE2), a vital protein in human cells, and then invigilated into target cells [4]. Of course, it must be said that not only the lungs are affected by the virus, other organs, such as the brain, can be targeted by it [5]. It has been shown that SARS-CoV-2 can enter the brain through the olfactory nerve and cause severe neuroinflammation [6]. With the entry of the virus and stimulation of the human immune system, a series of pro-inflammatory cytokines, such as interleukin (IL)-6, IL-1b, IL-8, IL-2R, and tumor necrosis factor (TNF-a), are produced and lead to cytokine storm [7]. In addition, hyperinflammatory reactions cause endothelial dysfunction [8] and pyroptosis [9].

By activation of different immune cells, several regulatory factors are also produced to control immunological events [10]. One of these regulators is MicroRNAs (miRNAs), which are small endogenous RNA molecules and control various physiological processes in cells [11]. Researchers had previously shown that the expression of some miRNAs is associated with respiratory syndromes and viral infections [12, 13]. In the case of COVID-19 disease, some researchers have compiled a list of up/down-regulated miRNAs by both bioinformatics [14–17] and experimental analysis [18–20]. The role of miRNAs in viral infections is very important and they can cause or modulate appropriate immune responses. Interestingly, some miRNAs can bind to viral mRNAs and block the expression of viral genes [21].

In this study, we evaluated the expression of four up/down-regulated inflammatory miRNAs and their mRNA targets in the blood of COVID-19 patients with different grades. We also wanted to see how the relative expression of these miRNAs and their mRNA targets differs during hospitalization. It is important to know the profile of miRNAs in COVID-19 because it can be useful in diagnosis and prognosis. In addition, new molecular drugs can be designed by miRNA profile [22].

## Materials and methods

### Bioinformatics

DIANA tools v.5.0 (http://diana.imis.athena-innovation.gr) was applied to identify miRNAs potentially related to COVID-19 and inflammatory pathways. Gene targets of the selected miRNAs were detected by miRDB (http://mirdb.org/) [23] and miRTarBase v.8.0 (http://mirtarbase.cuhk.edu.cn) [24], based on a target score. To find signaling pathways of the selected miRNAs, Kyoto Encyclopedia of Genes and Genomes (KEGG) [24] was used by *P *value (*P * <  0.05) [25].

### COVID-19 patients

In this cross-sectional study, 5 mL of blood sample were taken from COVID-19 patients, obtained from several health centers of Yazd, Tehran, and Zahedan province of Iran. Sampling was from December 20, 2020 to March 2, 2021. We also took blood samples during the patients’ hospitalization to see how the relative expression of miRNAs and mRNAs changed during hospitalization. The age range of patients was 38–63 years old and 52  ±  2% of them was female and 48  ±  2% was male. In this study, COVID-19 patients with grade 1 (*n*  =  21), grade 2 (*n*  =  20), grade 3 (*n*  =  20), grade 4 (*n*  =  21), and grade 5 (*n*  =  21) with informed consent were enrolled in the study. Positive real time-PCR, CT scan (depend on its grade), and clinical symptoms of COVID-19 were important inclusion criteria. Also, COVID-19 patients with severe comorbidities and inflammatory autoimmune diseases were excluded from this study. All COVID-19 patients were treated with remdesivir and favipiravir during hospitalization. Blood samples were also taken from 20 healthy individuals for control. All experiments were under the guidelines of the National Institute of Health, and the ethics committee of Zahedan University of Medical Sciences, Zahedan, Iran. (Ethical Code: IR.ZAUMS.REC.1399.316). After collecting peripheral blood samples, their serum was separated and stored at − 80 °C.

### The relative expression of miRNAs and mRNAs

Briefly, total RNAs were extracted from serum samples by mirPremier microRNA isolation kit (Sigma-Aldrich, USA), according to manufacture protocol. After confirmation of RNA purity by NanoDrop ND-1000 UV–VIS spectrophotometer, cDNA was synthesized by Mir-X miRNA First-Strand Synthesis kit (Takara Bio Inc., USA), according to manufacture protocol. Then, real time-PCR was done in presence of Mir-X miRNA qPCR SYBR (Invitrogen, UK). Finally, the threshold cycle (CT) values were recorded for each miRNA and mRNA. The relative expression of miRNAs and mRNAs was calculated by delta-delta CT formula. The reference gene for miRNAs was *RNU 48* and the reference gene for mRNAs was *GAPDH*.

### Statistical analysis

All data were reported as the mean ± standard deviation. To find significant differences between groups, a one-way ANOVA was used. Also, Spearman’s correlation coefficient was used to correlate the expression of miRNAs and their mRNA targets. A *P *value of less than 0.05 was considered statistically significant.

## Results

### Computational analysis

**Fig. 1:**
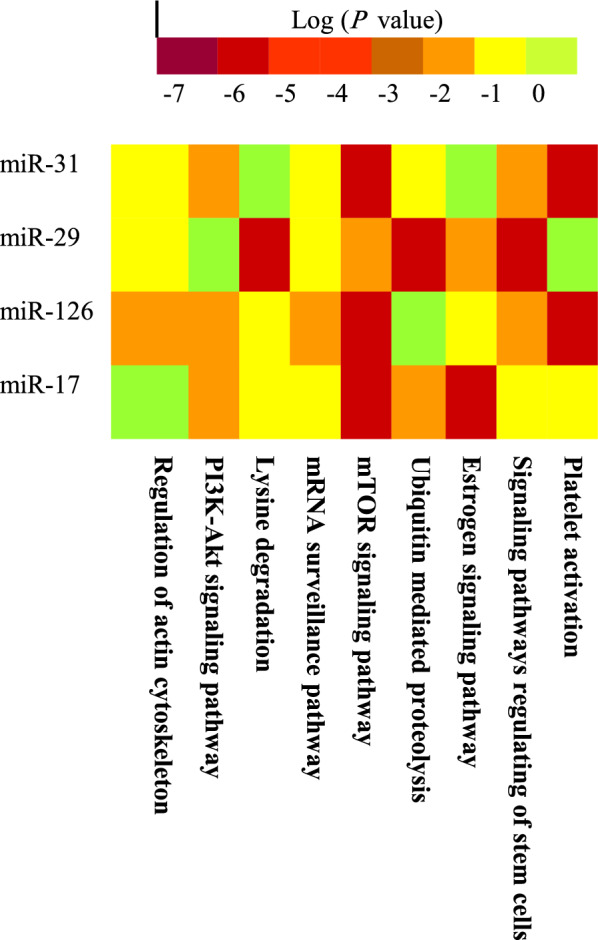
The heat map of significant pathways for each selected miRNA, extracted from the KEGG molecular pathway

Five target genes for each miRNA with the highest target score are shown in Table 1, obtained from miRDB and miRTarBase. For hsa-miR-31-3p, the best target gene was zinc finger MYM-type containing 5 (*ZMYM5*). For hsa-miR-29a-3p, the best target gene was collagen type V alpha 3 chain (*COL5A3*). For hsa-miR-126-3p, the best target gene was calmodulin-regulated spectrin-associated protein 1 (*CAMSAP1*). For hsa-miR-17-3p, the best target gene was dicer 1, ribonuclease III (*DICER1*). Figure 1 shows significant pathways for each miRNA, extracted from KEGG molecular pathway. As seen, important cellular pathways are Wnt signaling pathway and AMPK signaling pathway, PI3K–Akt signaling pathway, mRNA surveillance pathway, MAPK signaling pathway, and mTOR signaling pathway.

**Table 1:** The human gene targets of selected miRNAs, obtained from miRDB and miRTarBase

Target score	miRNA name	Gene symbol	Gene description
100	hsa-miR-31-3p	*ZMYM5*	Zinc finger MYM-type containing 5
99	hsa-miR-31-3p	CHMP4B	Charged multivesicular body protein 4B
99	hsa-miR-31-3p	ELOC	Elongin C
99	hsa-miR-31-3p	RXFP1	Relaxin family peptide receptor 1
98	hsa-miR-31-3p	HAUS4	HAUS augmin like complex subunit 4
98	hsa-miR-29a-3p	COL5A3	Collagen type V alpha 3 chain
97	hsa-miR-29a-3p	COL5A1	Collagen type V alpha 1 chain
97	hsa-miR-29a-3p	TET1	Tet methylcytosine dioxygenase 1
97	hsa-miR-29a-3p	HBP1	HMG-box transcription factor 1
96	hsa-miR-29a-3p	FBN1	Fibrillin 1
99	hsa-miR-126-3p	CAMSAP1	Calmodulin regulated spectrin-associated protein 1
99	hsa-miR-126-3p	PTPN9	Protein tyrosine phosphatase, non-receptor type 9
99	hsa-miR-126-3p	SLC7A5	Solute carrier family 7 member 5
98	hsa-miR-126-3p	PIK3R2	Phosphoinositide-3-kinase regulatory subunit 2
98	hsa-miR-126-3p	ADAM9	ADAM metallopeptidase domain 9
99	hsa-miR-17-3p	DICER1	Dicer 1, ribonuclease III
98	hsa-miR-17-3p	RAP2A	RAP2A, member of RAS oncogene family
98	hsa-miR-17-3p	KAT7	Lysine acetyltransferase 7
98	hsa-miR-17-3p	PTCHD1	Patched domain containing 1
98	hsa-miR-17-3p	COCH	Cochlin

### The expression of selected miRNAs and their mRNA targets

**Fig. 2:**
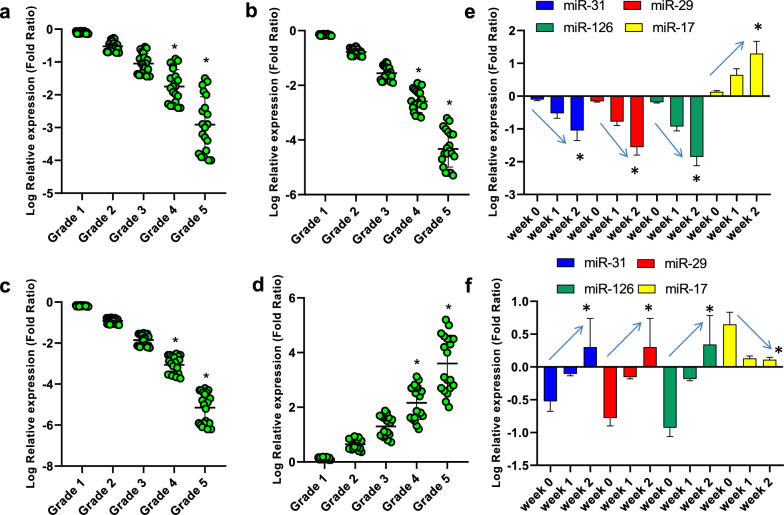
The relative expression of hsa-miR-31-3p (**a**), hsa-miR-29a-3p (**b**), hsa-miR-126-3p (**c**), and hsa-miR-17-3p (**d**) in COVID-19 patients with grades 1–5. *Indicates significant difference when compared with grades 1–3 by one-way ANOVA. The relative expression of hsa-miR-31-3p, hsa-miR-29a-3p, hsa-miR-126-3p, and hsa-miR-17-3p in COVID-19 patients who did not respond to treatment (**e**) and in COVID-19 patients who respond to treatment (**f**) during hospitalization. *Indicates significant difference when compared with week 0 by one-way ANOVA. Arrows indicate the trend of miRNA during hospitalization

Figure 2 shows the relative expression of hsa-miR-31-3p (a), hsa-miR-29a-3p (b), hsa-miR-126-3p (c), and hsa-miR-17-3p (d) in COVID-19 patients with grades 1–5. As found, the relative expression of hsa-miR-31-3p, hsa-miR-29a-3p, and hsa-miR-126-3p was significantly decreased with the increase of disease grade. Conversely, the relative expression of hsa-miR-17-3p was significantly increased with the increase of disease grade. These results were in consistent with the relative expression of these miRNAs during hospitalization. That is, in COVID-19 patients who did not respond to treatment (e), the relative expression of hsa-miR-31-3p, hsa-miR-29a-3p, and hsa-miR-126-3p was significantly decreased and the relative expression of hsa-miR-17-3p was significantly increased during hospitalization. Conversely, in COVID-19 patients who respond to treatment (f), the relative expression of hsa-miR-31-3p, hsa-miR-29a-3p, and hsa-miR-126-3p was significantly increased and the relative expression of hsa-miR-17-3p was significantly decreased during hospitalization.

**Fig. 3:**
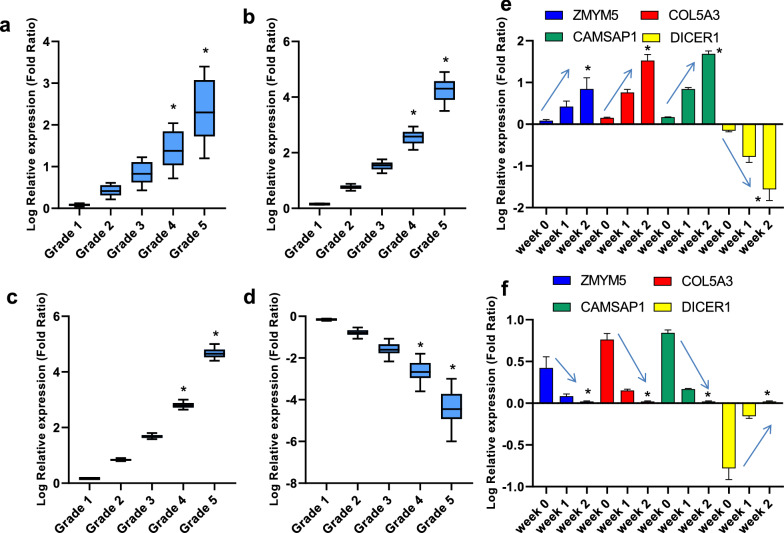
The relative expression of *ZMYM5* (**a**), *COL5A3* (**b**), *CAMSAP1* (**c**), *DICER1* (**d**) in COVID-19 patients with grades 1–5. *Indicates significant difference when compared with grades 1–3 by one-way ANOVA. The relative expression of *ZMYM5*, *COL5A3*, *CAMSAP1*, *DICER1* in COVID-19 patients who did not respond to treatment (**e**) and in COVID-19 patients who respond to treatment (**f**) during hospitalization. *Indicates significant difference when compared with week 0 by one-way ANOVA. Arrows indicate the trend of mRNA during hospitalization

Figure 3 demonstrates the relative expression of *ZMYM5* (a), *COL5A3* (b), *CAMSAP1* (c), *DICER1* (d) in COVID-19 patients with grades 1–5. As seen, the relative expression of *ZMYM5*, *COL5A3*, and *CAMSAP1* was significantly increased with the increase of disease grade. Conversely, the relative expression of *DICER1* was significantly decreased with the increase of disease grade. In COVID-19 patients who did not respond to treatment (e), the relative expression of *ZMYM5*, *COL5A3*, and *CAMSAP1* was significantly increased and the relative expression of *DICER1* was significantly decreased during hospitalization. Conversely, in COVID-19 patients who respond to treatment (f), the relative expression of *ZMYM5*, *COL5A3*, and *CAMSAP1* was significantly decreased and the relative expression of *DICER1* was significantly increased during hospitalization. Based on Table 2, a negative significant correlation was seen between the expression of (1) hsa-miR-31-3p and *ZMYM5*, (2) hsa-miR-29a-3p and *COL5A3*, (3) hsa-miR-126-3p and *CAMSAP1*, and (4) hsa-miR-17-3p and *DICER1* in COVID-19 patients with any grade (*P*  <  0.05).

**Table 2:** The correlation between the relative expressions of selected miRNAs and their mRNA targets at different grades of COVID-19 patients

	Grade 1	Grade 2	Grade 3	Grade 4	Grade 5
hsa-miR-31-3p					
*ZMYM5*	*R* = 0.72, *P * = 0.01	*R* = 0.82, *P * = 0.012	*R* = 0.84, *P * = 0.014	*R* = 0.91, *P * = 0.013	*R* = 0.94, *P * = 0.005
hsa-miR-29a-3p					
* COL5A3*	*R* = 0.71, *P * = 0.012	*R* = 0.85, *P * = 0.03	*R* = 0.88, *P * = 0.025	*R* = 0.9, *P * = 0.012	*R* = 0.93, *P * = 0.004
hsa-miR-126-3p					
* CAMSAP1*	*R* = 0.65, *P * = 0.016	*R* = 0.69, *P * = 0.032	*R* = 0.74, *P * = 0.03	*R* = 0.84, *P * = 0.019	*R* = 0.92, *P * = 0.002
hsa-miR-17-3p					
* DICER1*	*R* = 0.67, *P * = 0.025	*R* = 0.71, *P * = 0.045	*R* = 0.79, *P * = 0.023	*R* = 0.88, *P * = 0.02	*R* = 0.95, *P * = 0.001

## Discussion

This study aimed to evaluate the expression of four inflammatory miRNAs and their mRNA targets in the serum samples of COVID-19 patients with different grades. Also, we investigated the relative expression of these miRNAs and their mRNA targets during hospitalization. Since some COVID-19 patients respond to medication (e.g., remdesivir and favipiravir) and some of them do not respond well, the relative expression of miRNAs and their mRNA targets is different in the two groups. The first question is that which miRNAs must be selected and investigated from the human miRNA pool. Although it is a very difficult and complex question, there are two main solutions. The first solution is the evaluation of all human miRNAs with a microarray [26]. With this technique, although we can prepare a complete list of up/down-regulated miRNAs, it requires a complex and expensive device. Another solution is the selection of potential miRNAs using computational and bioinformatics methods. Most of the articles on miRNAs and COVID-19 have followed the same procedure [14–17]. Generally, miRNAs can be isolated from tissue [18] or blood samples [20]. Importantly, the miRNAs detected in the blood can help us to diagnose and prognoses COVID-19. Arguably, the main source of these miRNAs is from immune and respiratory cells. It is good to say that there are various destructive enzymes in the blood and the desired miRNAs may be rapidly broken down in it [27]. It is interesting to note other types of RNA, such as long non-coding RNAs (long ncRNAs, lncRNA), can also give us information about the presence of SARS-COV-2 [28].

In this study, we found that the relative expression of hsa-miR-31-3p, hsa-miR-29a-3p, and hsa-miR-126-3p was significantly decreased and the relative expression of their mRNA targets (*ZMYM5*, *COL5A3*, and *CAMSAP1*) was significantly increased with the increase of disease grade. Conversely, the relative expression of hsa-miR-17-3p was significantly increased and its mRNA target (*DICER1*) was significantly decreased with the increase of disease grade. This pattern was exactly seen during hospitalization of COVID-19 patients who did not respond to treatment. In COVID-19 patients who responded to treatment, the expression of selected miRNAs and their mRNA targets returned to the normal level. A negative significant correlation was seen between the expression of (1) hsa-miR-31-3p and *ZMYM5*, (2) hsa-miR-29a-3p and *COL5A3*, (3) hsa-miR-126-3p and *CAMSAP1*, and (4) hsa-miR-17-3p and *DICER1* in COVID-19 patients with any grade (*P*  <  0.05) and during  hospitalization. Our findings are consistent with the results of other researchers. For example, Farr et al. found that the expression of miR-31 was altered in COVID-19 [29]. Centa et al. [18] showed that hsa-miR-29a-3p is associated with endothelial dysfunction in post-mortem lung biopsies of COVID-19 patients. Widiasta et al. [30] indicated the potential role of ACE2-related microRNAs (such as hsa-miR-126) in COVID-19-associated nephropathy. Li et al. [31] declared that the differential miRNA expression (such as hsa-miR-17-3p) found in COVID-19 patients could regulate the immune system. Based on review literature, all four selected miRNAs have an important role in inflammation. For example, Shi et al. [32] found that miR-31 could mediate inflammatory signaling. Eken et al. [33] indicated that miR-29b could modulate the chronic inflammatory response. Tang et al. [34] showed that miR-126 could suppress inflammation in endothelial cells. Tan et al. [35] found that inhibition of miR-17 reduces the inflammation. Among the limitations of this project was that since only four miRNAs have been analyzed, we missed other important miRNAs. Therefore, it is suggested that in another study, the expression of other inflammatory miRNAs be evaluated in COVID-19 patients.

## Conclusions

Taken together, the relative expression of hsa-miR-31-3p, hsa-miR-29a-3p, and hsa-miR-126-3p was down-regulated and the relative expression of their mRNA targets was up-regulated with the increase of COVID-19 grade. This pattern was exactly seen during hospitalization for COVID-19 patients who did not respond to treatment. In COVID-19 patients who responded to treatment, the expression of selected miRNAs and their mRNA targets returned to the normal level. On the other hand, the relative expression of hsa-miR-17-3p was up-regulated and its mRNA target (*DICER1*) was down-regulated with the increase of COVID-19 grade.

## Data Availability

Not applicable.
